# Do Media Use and Physical Activity Compete in Adolescents? Results of the MoMo Study

**DOI:** 10.1371/journal.pone.0142544

**Published:** 2015-12-02

**Authors:** Sarah Spengler, Filip Mess, Alexander Woll

**Affiliations:** 1 Technische Universität München, Department of Sport and Health Sciences, München, Germany; 2 Karlsruhe Institute of Technology, Institute of Sports and Sports Science, Karlsruhe, Germany; Leibniz Institute for Prevention Research and Epidemiology (BIPS), GERMANY

## Abstract

**Purpose:**

The displacement hypothesis predicts that physical activity and media use compete in adolescents; however, findings are inconsistent. A more differentiated approach at determining the co-occurrence of physical activity and media use behaviors within subjects may be warranted. The aim of this study was to determine the co-occurrence of physical activity and media use by identifying clusters of adolescents with specific behavior patterns including physical activity in various settings (school, sports club, leisure time) and different types of media use (watching TV, playing console games, using PC / Internet).

**Methods:**

Cross-sectional data of 2,083 adolescents (11–17 years) from all over Germany were collected between 2009 and 2012 in the Motorik-Modul Study. Physical activity and media use were self-reported. Cluster analyses (Ward’s method and K-means analysis) were used to identify behavior patterns of boys and girls separately.

**Results:**

Eight clusters were identified for boys and seven for girls. The clusters demonstrated that a high proportion of boys (33%) as well as girls (42%) show low engagement in both physical activity and media use, irrespective of setting or type of media. Other adolescents are engaged in both behaviors, but either physical activity (35% of boys, 27% of girls) or media use (31% of boys and girls) predominates. These adolescents belong to different clusters, whereat in most clusters either one specific setting of physical activity or a specific combination of different types of media predominates.

**Conclusion:**

The results of this study support to some extent the hypothesis that media use and physical activity compete: Very high media use occurred with low physical activity behavior, but very high activity levels co-occurred with considerable amounts of time using any media. There was no evidence that type of used media was related to physical activity levels, neither setting of physical activity was related to amount of media use in any pattern.

## Introduction

Being physically active is an established protecting factor for chronic diseases. For instance, regular physical activity (PA) in young people has been associated with lower risk of overweight and cardiovascular disease risk factors and shown to influence health in adulthood [1, 2]. In contrast, high media use increases the risk of overweight in children and adolescents [3–5]. Further, PA and media use in young people are associated with adult behavior because physical activity and (media-based) inactivity track from adolescence into later life [1, 6]. However, to date it is not yet clear if and how PA and media use in adolescents are related to each other, or to what extent they are [7].

The displacement hypothesis [5, 8, 9] predicts that PA and media use compete in adolescents; however, to date findings are inconsistent. A review [10] reported no evidence to suggest that sedentary behavior (playing digital games, using a computer and watching TV) competes with physical activity in children and adolescents. A meta-analysis including 52 studies on children and adolescents [5] observed weak inverse relationships between PA and watching TV as well as between PA and playing computer or video games. The authors concluded that “claims that TV viewing, playing video games or using computers displace physical activity receive very limited empirical support” [5]. Conversely, other studies reported relevant moderate inverse relationships between PA and media use in adolescents [11–13], and one of these studies included a representative sample of adolescents in Germany [13]. In addition, some studies focusing on the relationship between PA, media use and health outcomes indicate that especially PA in sports clubs may counteract the adverse effect of sedentary behavior on certain health parameters [14, 15], whereas another study [16] showed that PA did not attenuate the obesity risk of TV viewing in youth. Overall, results on the interdependence of PA and media use in adolescents are inconsistent.

A more differentiated approach at determining the co-occurrence of PA and media use within subjects may be warranted. One possible approach is to not only look at correlations over the entire sample but to focus on different groups of adolescents who have specific patterns of behaviors. In this context a pattern represents a specific constellation of PA and media use within one subject. Moreover, considering different settings of PA and different media rather than using measures of overall PA and media use may be promising. Adolescent PA includes a complex combination of school-based PA, organized PA in sports clubs and leisure time PA [5, 17]. Especially in Germany—where sports clubs have a long tradition and are a popular place for being regularly physically active [18]—considering different settings of PA may be important. Further, total amount of media use comprises individually different amounts of time spent on watching TV, playing console games or using the PC / internet. For instance, in a recent study [19] using the computer clustered with high sports level at school and in clubs, while watching TV clustered with low sports participation, suggesting that assessing the use of different media separately is important [19]. However, complex analyses considering different settings of PA and / or types of media use are scarce.

A review from 2014 identified eight studies explicitly examining the clustering of PA and sedentary behavior / media use in children and adolescents by using empirical data-driven methodologies [20]. In addition, we identified another study [21] not included in the review. Five of these studies generated patterns for boys and girls separately [9, 21–24]. Operationalization of PA and sedentary behavior and the number of included variables differed between studies. A current representative study for Germany [13] provided data on the association between PA and media use in adolescents and authors concluded, that specific patterns should be examined with analytic statistical methods like cluster analysis. Furthermore, because the prevalence of PA and media use differs between boys and girls [18, 25], patterns should be examined for each sex. To the best of our knowledge, to date there is no study examining specific patterns of PA and media use in German children and adolescents.

The aim of this study was to determine the co-occurrence of PA and media use within subjects in adolescent boys and girls by identifying clusters with specific behavior patterns including PA in the settings school, sports club and leisure time and media use including watching TV, playing console games and using PC / Internet. Furthermore, the aim was to examine age and socio-economic status (SES) of the clusters.

## Methods

### Study design and participants

Data were collected between 2009 and 2012 at 167 study locations in all of Germany. They were part of the first data collection wave of the MoMo Study (Motorik-Modul) [26], which is a sub-study of the KiGGS Survey (German Health Interview and Examination Survey for Children and Adolescents) [27]. The KiGGS Survey as well as the MoMo Study are longitudinal studies that started in 2003 and will continue through 2020. Participants of the MoMo Study were randomly recruited from the KiGGS sample, allowing for the inclusion of data assessed in the KiGGS Survey. Cross-sectional data for the current study were taken from the first data collection wave. Study procedures were approved by the Federal Office for Data Protection and by the ethics committee of the University of Konstanz, Germany. Each parent and participant gave informed written consent before enrolment into the survey. The survey was conducted in accordance with the Declaration of Helsinki. Further details on the concept of the KiGGS Survey and the MoMo Study can be found elsewhere [26, 28]. For the current study data of 2,247 adolescents from the MoMo sample aged 11 to 17 were used. Subjects with missing data on relevant variables, i.e. PA at school, in sports clubs or leisure time and use of TV, console games or PC / internet were excluded. As the cases with missing data were completely at random (MCAR), their exclusion was allowed. Furthermore, three outliers were identified with single linkage cluster analysis and were excluded due to implausible data. Hence, 2,083 subjects remained for further analyses. 49.5% were male and the overall mean age was 13.8±1.9 years.

### Measures

#### PA and media use

The MoMo physical activity questionnaire (MoMo-PAQ) [18] captures weekly duration of PA in each of the following settings: elective PA at school (excluding compulsive physical education lessons), PA at sports clubs and leisure time PA outside of sports clubs. Participants could mention up to four different types of sports in each setting with an open answer format. For example in the sports club setting, if a participant engaged in a tennis club, a soccer club and a skiing club, he was asked to mention weekly duration of each of these types of sports. Further, for leisure time and sports clubs PA the months were recorded in which specific PA was performed to consider that some types of sports might not be performed the entire year, e.g. skiing. Using this information, PA indices for each of the three settings were calculated reflecting the time spent on PA in each setting in hours per week (duration*months performed/12 for each type of sport; sum score was built for each setting). Everyday PA, e.g. walking for transport, housework, gardening, was assessed separately. It was not included, as we decided to especially focus on voluntary PA assuming that voluntary PA depends on interests / priorities of the adolescents, rather than on duties and / or habits. Therefore the clusters rather represent adolescents’ choices. Further, validity and reliability of the questions asked towards everyday PA are quite low [29]. Reliability of the MoMo-PAQ ranges from low (k = 0.54 for everyday PA) to very good (k = 0.81 for sports club and leisure time PA) on item level. Validity is low with a significant correlation of r = 0.29 between overall activity index and accelerometer Actigraph GT1X data (Actigraph LLC, Pensacola, FL, USA). Thus, reliability and validity of the MoMo-PAQ are similar to those of other PA questionnaires for adolescents [29].

Media use was assessed by telephone interview in the KiGGS survey by asking adolescents about the daily amount of time they normally spend on watching TV or video, using a computer and playing console games. Answers were coded according to Lampert et al. [30]: “never” = 0 “up to 1 hour” = 0.5; “up to 2 hours” = 1.5; “up to 3 hours” = 2.5; “up to 4 hours” = 3.5; “5 or more hours” = 5. While PA was specified in hours per week, media use was specified in hours per day as these are the respective established dimensions of the two measures.

#### Age and socioeconomic status

Based on information on adolescents’ date of birth and date of the interview, age of adolescents was calculated. For the examinations we built two age groups: younger adolescents aged 11 to 13 years and older adolescents aged 14 to 17 years.

Information on parental education, professional status and net household income formed the basis of classification into low, medium and high SES groups. In each dimension, the highest available score of parental information was included and adolescents were classified according to their household’s status [31].

### Data analyses

All statistical tests were conducted using SPSS statistical software for Windows Version 22.0 (IBM Corporation, Armonk, NY, USA). Descriptive statistics were used to summarize socio-demographic variables, physical activity and media use. Socio-demographic differences were examined using Mann-Whitney tests for sex, and age group and Kruskal-Wallis tests for SES.

Patterns of PA and media use were identified using cluster analyses. The six variables “hours of PA at school per week”, “hours of PA in sports clubs per week” “hours of PA in leisure time per week”, “hours watching TV per day”, “hours playing console games per day” and “hours using a computer per day” were included in the analyses. Separate analyses were carried out for the entire sample and for each sex. Sex-specific analyses partly identified unique sex-specific clusters and further revealed several differences between clusters of boys and girls. Before clustering, data were standardized with z-scores. First, a single-linkage cluster analysis based on squared Euclidean distances was performed to identify outliers [32]. As a next step Ward’s method based on squared Euclidean distances was conducted. The empirically funded decision on optimal number of clusters was reached by visual inspection of the dendogram, investigating the increase of error sum of squares and comparing explained variance of the variables between different cluster solutions. Based on these considerations, content interpretation was taken into account for the final decision which is requested for cluster analyses [32]. The chosen cluster solution was taken as starting partition for K-means analysis, which optimizes the classification. Reliability and stability of the solution was tested by repeating these analyses with a randomly selected subsample of 50%. Homogeneity was assessed as the percentage of consistently allocated participants in K-means analysis and by comparing mean distances of one element of a cluster to its center with distance of the center to the other cluster centers and comparing variance of an item within clusters with variance of the item within the entire sample. Chi-square tests were used to identify differences in age and SES between clusters. The significance level for all statistical tests was set a priori to α = .05 and adjusted using Bonferroni correction for the multiple Chi-square tests.

## Results

### Description of PA and media use

The mean time spent on different PA per week and media use per day is listed in Table 1. Significant differences (p < .01) between sexes were observed in all behaviors except in PC / Internet use, with boys having higher values than girls. All behaviors differed significantly between age groups (p < .05). Adolescents aged 14–17 years had higher values than adolescents aged 11–13 on all variables except voluntary PA at school. Further, values of PA in sports clubs did not differ relevantly between age groups. In addition, with regard to voluntary PA at school differences between age groups and sexes were in fact significant but too small to be relevant. No significant differences in voluntary PA at school and PC / Internet use were observed between SES groups. All other behaviors differed significantly between SES groups (p < .01), whereat the high SES group had higher values in PA and lower values in media use.

**Table 1:** Mean ± SD number of hours per week (PA) / day (media) spent on different leisure activities separated by socio-demographic variables.

		N	Voluntary PA school (h / week) ±SD		PA sports club (h / week) ±SD		PA leisure time (h / week) ±SD		TV (h / day) ±SD		Console games (h / day) ±SD		PC / Internet (h / day) ±SD	
**Sex** ^**a**^	**Male**	1031	0.4±1.0	**	2.1±2.2	**	1.4±2.4	**	1.7±1.2	**	0.8±1.0	**	1.3±1.3	
	**Female**	1052	0.3±0.8		1.5±2.0		0.9±1.7		1.4±1.1		0.2±0.5		1.3±1.2	
**Age group** ^**a**^	**11–13y**	953	0.4±0.9	**	1.8±1.9	*	1.0±1.9	**	1.4±1.1	**	0.5±0.7	**	0.9±1.0	**
	**14–17y**	1130	0.3±0.9		1.8±2.3		1.2±2.2		1.7±1.2		0.7±0.9		1.7±1.4	
**SES** ^**b**^	**Low**	196	0.4±0.9		1.3±2.1	**	1.0±2.5	**	1.9±1.3	**	0.7±1.0	**	1.5±1.5	
	**Medium**	1362	0.4±0.9		1.7±2.0		1.1±2.1		1.6±1.2		0.5±0.9		1.3±1.3	
	**High**	523	0.4±1.0		2.3±2.3		1.2±1.8		1.3±1.0		0.3±0.7		1.2±1.1	
**Total**		2083	0.4±0.9		1.8±2.1		1.1±2.1		1.6±1.2		0.5±0.8		1.3±1.3	

SES = socio-economic status

^a^ significance level examined using Mann-Whitney tests

^b^ significance level examined using Kruskal-Wallis tests

* p < .05

** p < .01

### Identification of clusters

**Fig 1:**
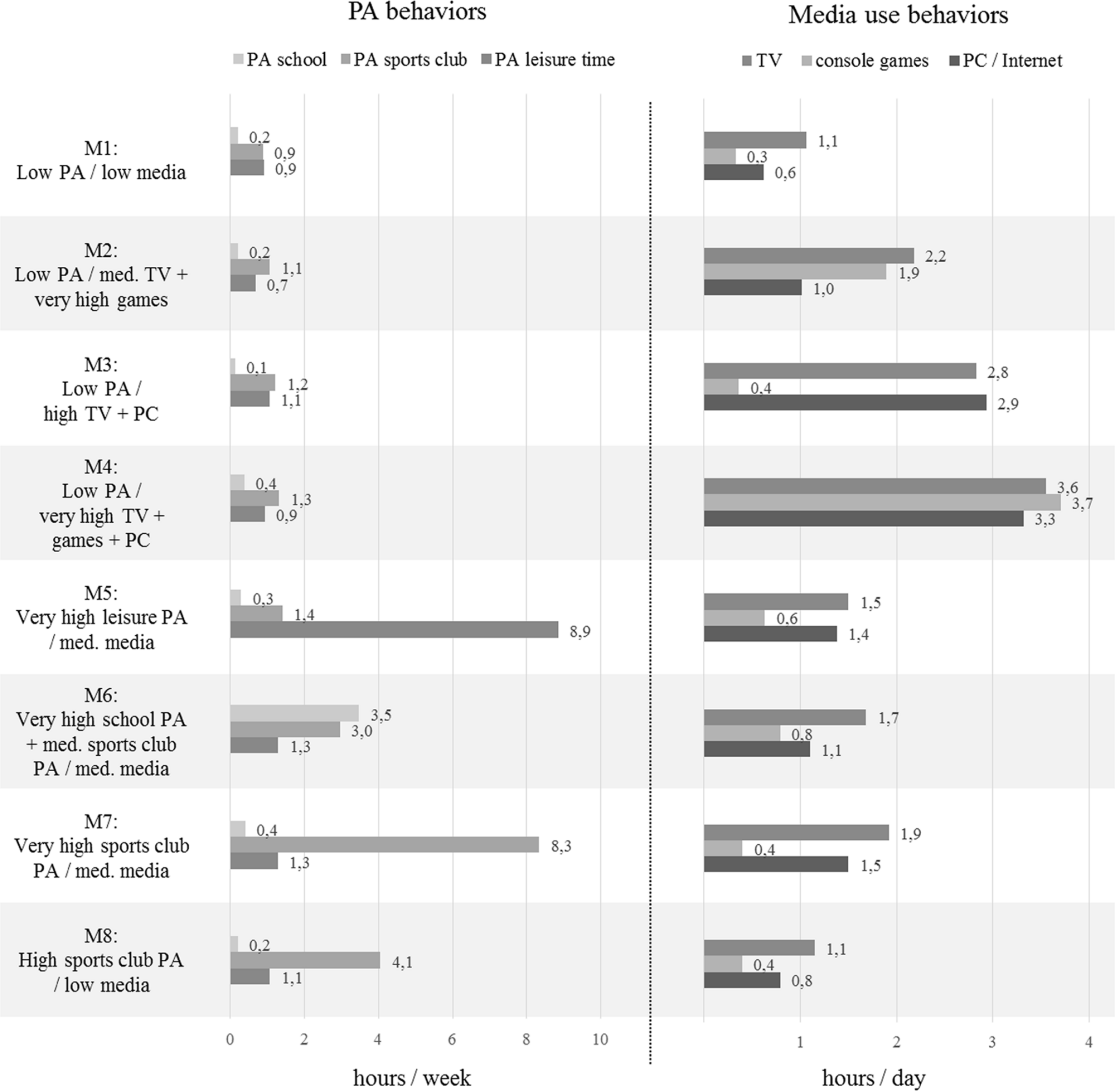
Mean hours of PA per week and media use (per day in boys’ clusters.

**Fig 2:**
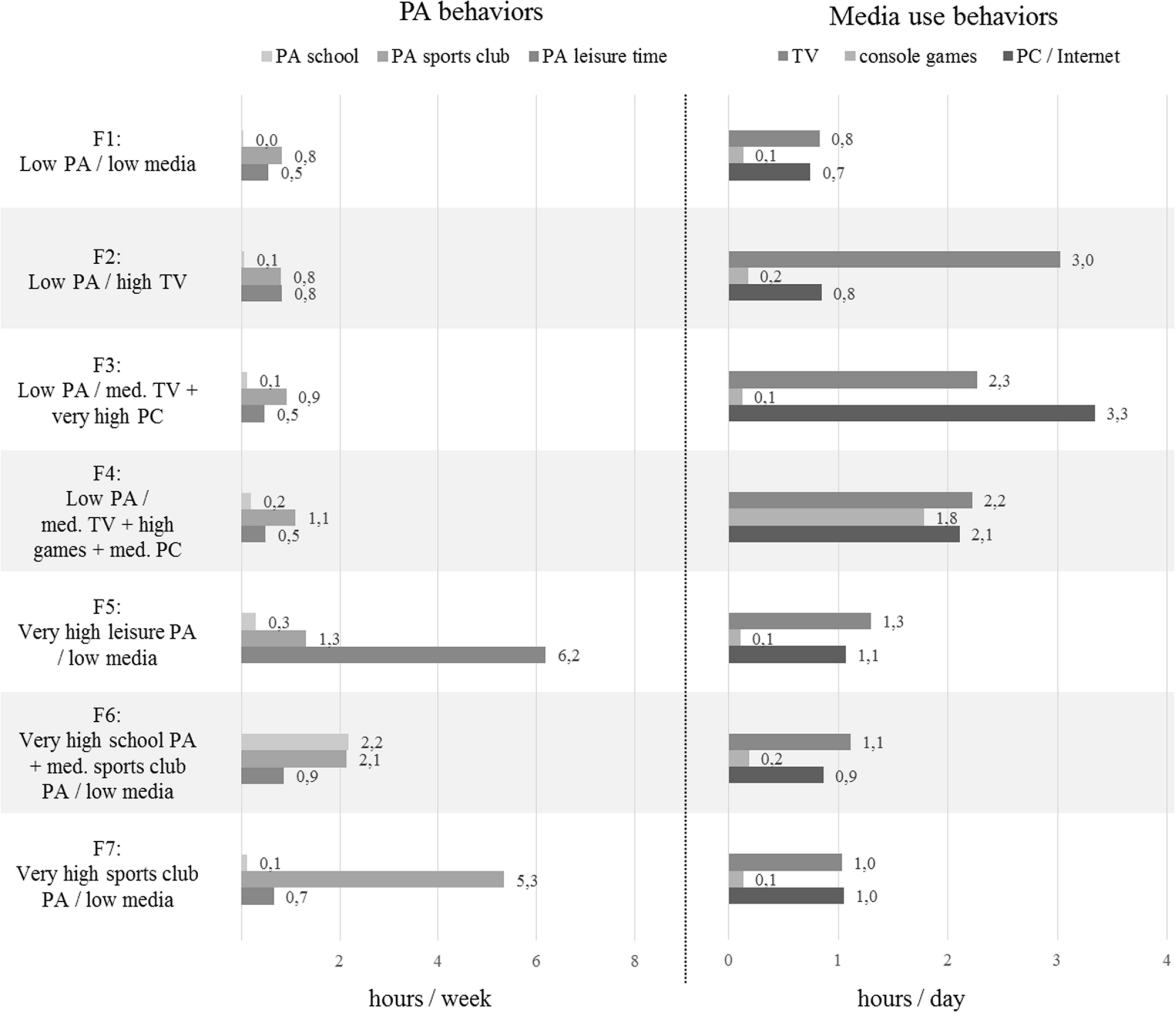
Mean hours of PA per week and media use per day in girls’ clusters.

Eight clusters were identified for boys (Fig 1) and seven clusters for girls (Fig 2). Stability tests of the solutions showed excellent to moderate agreement (Kappa = .88 in boys; Kappa = .67 in girls). Overall, homogeneity was achieved: 73.8% of boys and 83.9% of girls were consistently allocated to the same cluster. For both sexes, mean distances to cluster centers in each cluster were smaller than distances of the respective center to the other cluster centers. The variance of the items within clusters was satisfactorily small in most cases for boys and girls.

To describe the PA levels of the clusters in the three settings and the amount of time using TV, console games and PC / Internet, we used the labels “low” (lower than mean), “medium” (between mean and mean + 1 SD), “high” (higher than mean + 1 SD but not exceeding mean + 1.5 SD) and “very high” (exceeding mean + 1.5 SD), which are related to the mean of each variable of the total sample (see Table 1).

In boys, one cluster was identified with low PA as well as low media use. One third of all boys belong to this cluster (M1) (Table 2). Three other clusters (M2, M3, M4) have high values in overall media use and low overall PA levels, but the type of media they use is different. M2 has medium values in watching TV and very high values in playing console games and a rather low level of PC / internet use combined with low PA levels. M3 has high values in watching TV and PC / internet use but low values in playing console games combined with low PA levels, while in M4 all three media are used on a very high level and PA levels are again low. M5, M6 and M7 have high PA levels and a medium media use, compared to the other clusters. In these three high PA clusters the setting of PA differs. M5 has very high PA levels in leisure time, but low PA levels in the other two settings combined with medium values in all three types of media. M6 has very high levels of voluntary PA in the school setting, medium values in the sports clubs setting and low PA levels in leisure time. Also in this cluster values in each type of media are medium. M7 has a very high level of PA in the sports clubs setting, a low level of PA in the school setting, a medium level in the leisure time setting and medium values in each type of media. M8 is a cluster with rather high sports club PA, a low PA level in school, a medium PA level in leisure time and rather low overall media use. Table 2 shows the z-scores of the final cluster solution in boys as well as socio-demographic correlates.

**Table 2:** Z-scores of the final cluster solution in boys and socio-demographic correlates.

		M1		M2	M3	M4	M5	M6	M7	M8	Total	Sig.
**N (%)**		343 (33.3)		126 (12.2)	147 (14.3)	50 (4.8)	53 (5.1)	65 (6.3)	50 (4.8)	197 (19.1)	1031 (100)	
**Z-score: PA at school**		-.22		-.21	-.29	-.05	-.12	3.06	-.02	-.22	0	
**Z-score: PA at sports club**		-.54		-.46	-.39	-.35	-.31	.39	2.78	.87	0	
**Z-score: PA at leisure time**		-.20		-.30	-.14	-.19	3.18	-.04	-.05	-.13	0	
**Z-score: TV use**		-.53		.42	.97	1.57	-.16	0	.19	-.46	0	
**Z-score: console games playing**		-.42		1.15	-.39	2.98	-.12	.04	-.35	-.35	0	
**Z-score: PC / Internet use**		-.52		-.21	1.32	1.63	.08	-.14	.18	-.38	0	
***Socio-demographics (%)***												
**Age group**	**11–13y**	60.9		41.3	22.4	26.0	45.3	49.2	24.0	51.3	46.2	**
	**14–17y**	39.1		58.7	77.6	74.0	54.7	50.8	76.0	48.7	53.8	
		^b^ ^c^ ^d^ ^f^		^a^ ^c^	^a^ ^b^ ^e^ ^g^	^a^ ^g^		^c^	^a^ ^g^	^c^ ^d^ ^f^		
**SES**	**Low**	8.2		14.3	13.6	12.0	7.7	6.2	12.0	6.6	9.6	**
	**Medium**	65.2		71.4	61.9	78.0	71.2	67.7	58.0	56.9	64.6	
	**High**	26.6		14.3	24.5	10.0	21.2	26.2	30.0	36.5	25.8	
				^g^		^g^				^b^ ^d^		

SES = socio-economic status

** p < .01

^a^ significantly different to M1 (p<0.0017)

^b^ significantly different to M2 (p<0.0017)

^c^ significantly different to M3 (p<0.0017)

^d^ significantly different to M4 (p<0.0017)

^e^ significantly different to M6 (p<0.0017)

^f^ significantly different to M7 (p<0.0017)

^g^ significantly different to M8 (p<0.0017)

In girls, similar to the boys, one cluster (F1) with low levels of both PA and media use was identified. The proportion of girls belonging to this cluster is 42.1% (Table 3). Another three clusters (F2, F3, F4) have low overall PA levels and high media use, while the use of the three types of media again differs between clusters. F2 has high values in watching TV, low values in playing console games and using PC / internet combined with low overall PA levels. F3 has medium values in watching TV and very high values in using PC / internet, but low values in playing console games, again combined with low overall PA levels. In F4 values in all media are medium to high combined with low overall PA levels. In contrast, clusters F5, F6 and F7 have high PA levels and low overall media use. F5 has very high PA levels in the leisure time setting, low PA levels in the school and sports clubs settings combined with low overall media use. F6 has very high levels of voluntary PA at school, medium levels of PA in sports clubs and low PA levels in leisure time, again combined with low overall media use. In F7 the sports club setting for PA predominates, levels of PA at school and in leisure time are low as well as media use is low. Table 3 shows the z-scores of girls’ final cluster solution and their socio-demographic correlates.

**Table 3:** Z-scores of the final cluster solution in girls and socio-demographic correlates.

		F1	F2	F3	F4	F5	F6	F7	Total	Sig.
**N (%)**		443 (42.1)	97 (9.2)	164 (15.6)	65 (6.2)	54 (5.1)	105 (10.0)	124 (11.8)	1052 (100)	
**Z-score: PA at school**		-.32	-.30	-.23	-.13	-.01	2.34	-.22	0	
**Z-score: PA at sports club**		-.37	-.37	-.32	-.23	-.11	.30	1.94	0	
**Z-score: PA at leisure time**		-.20	-.04	-.25	-.24	3.10	-.02	-.14	0	
**Z-score: TV use**		-.53	1.45	.76	.73	-.11	-.28	-.22	0	
**Z-score: console games playing**		-.23	-.13	-.24	3.18	-.27	-.12	-.22	0	
**Z-score: PC / Internet use**		-.44	-.37	1.62	.64	-.19	-.35	-.21	0	
***Socio-demographics (%)***										
**Age group**	**11–13y**	49.0	45.4	25.0	49.2	38.9	54.3	52.4	45.3	**
	**14–17y**	51.0	54.6	75.0	50.8	61.1	45.7	47.6	54.7	
		^c^	^c^	^a^ ^b^ ^d^ ^e^ ^f^	^c^		^c^	^c^		
**SES**	**Low**	6.8	17.5	11.6	16.9	11.1	7.6	4.8	9.2	**
	**Medium**	64.8	69.1	72.0	72.3	63.0	67.6	58.9	66.3	
	**High**	28.4	13.4	16.5	10.8	25.9	24.8	35.3	24.5	
		^b^ ^d^	^a^ ^f^	^f^	^a^ ^f^			^b^ ^c^ ^d^		

SES = socio-economic status

** p < .01

^a^ significantly different to F1 (p<0.0023)

^b^ significantly different to F2 (p<0.0023)

^c^ significantly different to F3 (p<0.0023)

^d^ significantly different to F4 (p<0.0023)

^e^ significantly different to F6 (p<0.0023)

^f^ significantly different to F7 (p<0.0023)

## Discussion

In this study, we investigated the co-occurrence of adolescents’ PA and media use by identifying specific behavior patterns including PA in the settings school, sports club and leisure time as well as media use including watching TV, playing console games and using PC / Internet. Patterns were identified separately for each sex.

In general, boys spent more time on both PA and media use than girls, which is in agreement with previous studies [24, 33]. Moreover, in line with the results of Lampert et al. [30] older adolescents spent more time on PC / internet. Adolescents with a low SES spent more time on media use and less on PA than adolescents in the higher status groups, which confirms results of other studies [13, 25]. Our cluster analyses resulted in an 8 cluster solution for boys and a 7 cluster solution for girls. In this study solutions with higher numbers of clusters were preferred because these solutions reflected finely graduated patterns considering different settings of PA and the use of different media. The results showed that a high proportion of adolescents show low engagement in both physical activity and media use, irrespective of setting or type of media. Other adolescents are engaged in both behaviors, but either physical activity or media use predominates. Here, specific patterns of boys and girls will be discussed separately and compared with each other. We will focus on indications with regard to a possible competition of media use and PA and discuss notable results regarding single patterns and socio-demographic correlates.

In boys, we identified eight patterns. Every single pattern has its own characteristics. Except from M1, where both PA and media use are low, each pattern is predominated by either PA or media use. Three patterns can be summarized as high media use patterns (M2, M3, M4). Boys in these three high media use clusters all had low PA levels, irrespective of the types of media they preferred. This fact indicates that in boys no interdependence exists between the use of special types of media and the level of PA. These high media use clusters (M2, M3, M4) combined contain 31.3% of boys, which confirms results of Manz et al. [13] who reported that around 30% of boys use any type of media for more than five hours per day (N = 4,941; aged 11–17).

Three patterns in boys can be summarized as high PA patterns (M5, M6, M7). Each of the three clusters is predominated by PA in one specific setting, but the choice of PA setting is not associated with media use in boys, as media use was similar in all of the three clusters. These are particularly small clusters (4.8–6.3%) and contain combined 16.2% of boys. Comparable results have been reported by Gorely et al. [23], where 15.4% of boys belonged to a cluster which was dominated by high sports levels (N = 1,371; age 13–16). Only one other study identifying patterns [34] distinguished between different PA settings; however in that study a three cluster solution was preferred and therefore their cluster solution did not differentiate possible setting-specific patterns.

The largest boys’ cluster in our study (33%) was M1 which included boys with low PA and low media use. Such a cluster was also identified in previous studies with comparable cluster sizes [21, 22]. 19.1% of boys belonged to M8 showing a pattern of higher sports club PA compared to M1 and low media use. M1 and M8 had similar characteristics except that boys in M1 had a low overall PA level and boys in M8 had a rather high sports club PA level. Interestingly, M1 did not show a noteworthy SES distribution, but M8 had a high prevalence of boys with a high SES. Thus, M8 seems to represent a very typical pattern for boys with a high SES.

The identified clusters for boys revealed that patterns with lowest media use (2 hours / day) also include low / moderate PA behavior (M1 / M8). Patterns with rather high—compared to current recommendations of two hours per day [35]–media use (3.5–3.8 hours / day) include high PA behavior (M5, M6, M7). Finally, in patterns with very high media use (>5 hours / day) low PA behavior co-occurred (M2, M3, M4). Similar results have been reported in another study [21] using accelerometers for measuring PA and sedentary time (N = 766; age 10–12). With regard to a possible competition of media use and PA these results let assume that high PA—regardless of setting—does not compete with media use but that very high media use—regardless of type of media—competes with PA. It is feasible that because time with high PA levels is possibly limited by physical exhaustion, there is still considerable time for using any media. In contrast, spending a lot of time on media—as in M4 —in addition to time spent at school and time spent sleeping leaves none to little time left for any PA [5]. Moreover, our results suggest that boys are involved in other leisure activities, e.g. music, arts, socializing behaviors, or further PA and media use behaviors which weren’t included in the analysis (e.g. housework, gardening, talking on the phone, listening to music) because almost one third of boys was neither highly engaged in PA nor in media use in our study. In a study by Gorely et al. [23], boys who were less engaged in PA and media use showed high levels of homework, which may be another concurring leisure activity for boys in M1. In comparison, clusters M2 to M8 indicate that while many adolescents engage in both PA and media use, one behavior predominates. A study including computer use, working, socializing behavior, TV / video viewing, homework and sports / exercise in their cluster analysis also reported the predominance of one behavior [23]. A closer look on boys’ particular patterns showed that boys tended to have either a high sports club PA level or a high leisure time PA level. Hence, also within PA behaviors one behavior (i.e. setting) may predominate. One possible reason may be that the decision to participate in a sports club / voluntary PA at school or to be physically active in leisure time might depend on special preferences concerning content and social environment of PA in each setting. Further, referring to the different motives of participating in PA [36], the level of interest in performance and competition may guide the decision of the PA setting.

In girls, we identified seven behavior patterns. Similar to those in boys, the patterns are either predominated by PA or media use or none of the included behaviors dominates. Three patterns can be summarized as media use patterns (F2, F3, F4) with smaller amounts of media use than in boys. This result is in line with our descriptive data (Table 1) and with results of previous national and international studies [13, 25, 37]. Within these media use patterns, we observed a classic pattern of TV watching behavior: girls in F2 spent three of four hours per day spent on media on watching TV. In this cluster, girls with low SES were overrepresented. A comparable cluster was not observed in boys because media-oriented boys seem to be interested in at least one other media besides TV. However, although watching TV predominated media use in F2, PA behavior was similar as in F3 and F4, indicating that also in girls no interdependence exists between the use of special types of media and the level of PA.

Three patterns in girls can be summarized as high PA patterns (F5, F6, F7) in relation to the other girls’ patterns, with fewer hours of PA than in corresponding patterns in boys. Girls in these three patterns all had low media use levels. This fact indicates again that an interdependence of the choice of specific PA settings and media use doesn’t exist.

The percentage of girls with a high PA pattern was higher than in previous studies (26.9% vs. 14.5% and 20.9%) [21, 23]. This discrepancy may have been caused by the different assessment methods used in these studies. In our study, girls with moderate activity levels were allocated to the PA-oriented clusters by the cluster analyses which may also explain the different percentages compared to boys’ high PA clusters (16.2%) where boys who were moderately physically active—related to other boys—were allocated to the additional cluster M8.

F1 was characterized by a pattern of low PA levels and low media use which has also been reported in the literature [9, 21–23]. Gorely et al. [23] showed that girls in this cluster were highly engaged in socializing activities which may have also been the case for girls in cluster F1 in our study. Furthermore, these girls might have been also engaged in activities such as music or arts. We observed a similar cluster in boys (M1). However, time spent on PA and on media in F1 was even lower than in M1.

In the context of a possible competition of media use and PA, occurrence of PA levels and media use in girls showed the same tendency as in boys: lowest media use occurred with low PA levels; low to moderate media use (compared to the other girls’ clusters) occurred with high PA levels; high media use occurred with low PA levels. However, overall levels of both PA and media use were lower in girls than in boys. Hence, in contrast to boys, girls with high media use should still have time for PA but seem to spend this time on other leisure activities than PA, e.g. music, arts, socializing activities. Overall, in girls other leisure activities seem to play an important role, as the proportion of girls in F1 (neither high PA nor high media use) was fairly high and overall PA and media use was lower than in boys.

This study was the first study in Germany using analytic statistical methods to determine the co-occurrence of PA behavior in various settings and the use of different types of media and hence contributes to understanding the interdependence among these behaviors in adolescents. However, results were based on questionnaire data and adolescents are known to tend to overestimate time spent on PA and some might underestimate time using any media because of desirability. Further, cluster analyses are explorative methods, and results are in part based on the investigators’ decisions. In addition, it is a subject-centered and date-driven approach and thus generalization of findings to other populations is limited. Hence, further studies are needed to augment evidence on the existence of the identified clusters. In addition, including other leisure activities into the analyses may provide a more comprehensive picture of leisure activity patterns and further elucidate the co-occurrence and interdependence of different leisure activity behaviors.

In summary, about two thirds of adolescents in Germany seem to be engaged in both PA and media use but either PA or media use appears to predominate. There was no evidence that type of used media was related to PA levels, neither setting of PA was related to amount of media use in any pattern. Furthermore, a considerable number of adolescents—both boys and girls—in Germany seem to be neither highly engaged in PA nor in media use indicating that other leisure activities are at least as important as PA or media use. The results of this study support to some extent the hypothesis that media use and PA compete: Very high media use—regardless of type of media—occurred with low PA behavior, but very high PA levels—regardless of setting—co-occurred with considerable amounts of time using any media, particularly in boys. For public health programs these results indicate the importance of addressing both media use and PA, as in two thirds of adolescents no interrelationship seems to exist. For future studies on a possible competition of media use and PA, the clusters of adolescents who combine very high media use with low PA would be of special interest. However, longitudinal studies are needed to give a final answer to the question of displacement.

## Supporting Information

S1 Dataset MoMo Study dataset. (XLSX)
