# Correction: Do Media Use and Physical Activity Compete in Adolescents? Results of the MoMo Study

**DOI:** 10.1371/journal.pone.0147093

**Published:** 2016-01-08

**Authors:** The PLOS ONE Staff

The Funding section is incorrect. The publisher apologizes for the error. The correct funding information is as follows: The study was funded by the German Bundesministerium für Bildung und Forschung (Federal Ministry of Education and Research) (Grant number: 01ER0810). The funders had no role in study design, data collection and analysis, decision to publish, or preparation of the manuscript.
